# Diagnostic Performance and Subcategorization of Hikikomori Cases in Oman: A Cross‐Sectional Study and Comparative Analysis of the HQ‐25 and HiDE‐I Assessment Tools

**DOI:** 10.1155/tswj/1094187

**Published:** 2026-04-05

**Authors:** Nutaila Al-Kharusi, Yahya M. Al-Farsi, Moon Fai Chan, Samir Al-Adawi, Nasser Al-Sibani

**Affiliations:** ^1^ Department of Family Medicine and Public Health, College of Medicine and Health Sciences, Sultan Qaboos University, P. O. Box 35 P. C. 123 Al-Khoudh, Seeb, Sultanate, Oman, squ.edu.om; ^2^ Department of Behavioral Medicine, College of Medicine and Health Sciences, Sultan Qaboos University, P. O. Box 35 P. C. 123 Al-Khoudh, Seeb, Sultanate, Oman, squ.edu.om

**Keywords:** classification thresholds, hide-i, hikikomori, HQ-25, Oman, ROC analysis

## Abstract

**Background:**

Hikikomori, a severe form of social withdrawal, has been predominantly studied in East Asia but remains underexplored in Middle Eastern contexts. As societal and cultural factors influence its manifestation, developing reliable diagnostic tools is critical for accurate identification and intervention. The Hikikomori Questionnaire‐25 (HQ‐25) serves as a self‐reported screening measure, while the Hikikomori Diagnostic Evaluation Interview (HiDE‐I) is used for clinical confirmation.

**Aim:**

This study aims to assess the diagnostic classification and predictive performance of the HQ‐25 compared to the HiDE‐I in an Omani sample, with a specific focus on refining cutoff thresholds for better classification accuracy.

**Method:**

A cross‐sectional study was conducted in Oman in 2024, enrolling 454 participants from clinical and community settings. Participants were classified as either patients (psychiatric service users) or attendees (nonclinical individuals). The HQ‐25 was administered at four cutoff thresholds (≥ 42, ≥ 50, ≥ 62, ≥ 75). The HiDE‐I was used as the clinical criterion standard, classifying cases as pathological, at‐risk, or resembling hikikomori. Diagnostic metrics—including sensitivity, specificity, predictive values, and receiver operating characteristic (ROC) curves—were calculated.

**Results:**

Table‐based analyses demonstrated that at the ≥ 42 cutoff, the HQ‐25 yielded 62.9% sensitivity and 57.6% specificity under the strict HiDE‐I definition, and 80.0% sensitivity with 53.4% specificity under the confirmed HiDE‐I definition. ROC analyses across all thresholds showed area under the curve (AUC) values ranging from 0.58 to 0.66 (strict HiDE‐I) and 0.55 to 0.85 (confirmed HiDE‐I), with the highest classification accuracy observed among psychiatric patients.

**Conclusion:**

The HQ‐25 is a useful screening tool but insufficient on its own for diagnosing hikikomori. Incorporating both diagnostic tiers revealed its limitations and reinforced the need for structured clinical assessments to improve accuracy, especially in nonclinical settings.

## 1. Introduction

Hikikomori, characterized by extreme social withdrawal and prolonged self‐imposed isolation, was initially recognized in Japan but has since been identified as a global phenomenon [1–5]. Cases have been reported across East Asia, Europe, the Middle East, and the Americas, highlighting the need for culturally sensitive diagnostic tools and intervention strategies [6, 7]. Despite this growing recognition, research on hikikomori remains scarce in the Middle East, particularly in Oman, where sociocultural and economic factors may shape its presentation differently from other regions [8].

Hikikomori is typically defined as prolonged social withdrawal lasting at least 6 months, accompanied by functional impairments and distress [9]. While often associated with school or work avoidance, its distinction from other psychiatric disorders remains debated [10, 11]. Studies suggest that economic instability, digital dependency, family dynamics, and mental health conditions contribute to its onset [12, 13]. Recent evidence indicates that social media and gaming may reinforce withdrawal behaviors, especially post‐COVID‐19, when digital interactions have become primary modes of social engagement [14–16].

In Oman, rapid social changes, increasing academic and career pressures, and evolving family structures may contribute to emerging hikikomori‐like behaviors [8, 17]. A recent study by Chan et al. [18] identified two distinct subgroups of individuals exhibiting hikikomori‐like idiom of distress (HLID) in Oman, highlighting the role of digital dependency, living alone, and mental health history in shaping social withdrawal. Psychological distress is a growing concern among young adults, particularly women, who experience higher rates of anxiety and depression [19]. However, stigma surrounding mental health often leads to underreporting and a lack of professional intervention, which may obscure the true prevalence of hikikomori in Oman, as stigma has been identified as a key barrier to mental health service utilization in the country [20, 21].

Oman, a high‐income Arab country in the Middle East, spans approximately 309,500 km^2^. As of 2023, its population is estimated to be around 5.1 million, with women comprising approximately 34.5% of the total population [22, 23]. Despite the economic development, mental health services remain underutilized due to stigma and limited awareness. A study in Oman found that 42.9% of participants identified stigma as a major barrier to seeking mental health care, with even higher rates among those with current mental health problems [21]. Additionally, research suggests that public attitudes toward mental illness in Oman are significantly influenced by sociodemographic factors, further reinforcing stigma and misconceptions. While the Hikikomori Questionnaire‐25 (HQ‐25) has been validated in Oman [8], further refinements and culturally specific adaptations are necessary to improve its diagnostic precision and fully assess the prevalence and impact of hikikomori in the region.

Efforts to refine hikikomori assessment tools have led to the development of structured diagnostic interviews such as the Hikikomori Diagnostic Evaluation Interview (HiDE‐I) and self‐report measures such as the HQ‐25 [14, 24]. While HQ‐25 is widely used, concerns remain about its potential for misclassification, as it may overidentify transient social withdrawal rather than clinically significant cases [25]. This underscores the need for a multimethod approach incorporating clinician‐administered assessments to improve diagnostic accuracy [11].

A key aspect of refining diagnostic accuracy involves evaluating screening tool performance using measures such as receiver operating characteristic (ROC) curve analysis, which assesses sensitivity and specificity across various cutoff points [1, 26]. Prior research indicates that psychiatric screening tools often prioritize sensitivity over specificity, leading to false positives [27]. Applying ROC analysis to hikikomori screening tools in Oman will help establish appropriate classification thresholds, ensuring accurate identification of affected individuals.

While this study is the first in Oman and the Arab region to assess the diagnostic utility of HQ‐25 in a clinical population and its agreement with HiDE‐I, prior research has already validated HQ‐25 in the Omani context [8, 18]. Al‐Sibani et al. [8] confirmed the factorial validity of HQ‐25 in Oman and explored the prevalence and associated factors of HLID in a general population sample. Their study provided initial psychometric validation but did not examine HQ‐25’s performance in a clinical setting or its concordance with a structured diagnostic interview. Chan et al. [18] further explored hikikomori subgroups in Oman but did not assess HQ‐25 against HiDE‐I.

This study extends previous findings by applying HQ‐25 in a psychiatric clinical population and comparing its classification accuracy with HiDE‐I through ROC curve analysis. By determining optimal cutoff thresholds, it aims to refine screening accuracy and enhance the specificity and sensitivity of hikikomori assessment in Oman. This distinction is crucial, as previous studies primarily focused on community‐based samples, whereas this study bridges the gap between self‐reported social withdrawal and clinician‐confirmed diagnoses.

## 2. Methods

### 2.1. Study Design and Setting

This cross‐sectional study was conducted at the Behavioral Medicine Clinic of Sultan Qaboos University Hospital (SQUH) in Muscat, Oman, between October 13, 2023, and October 8, 2024. In December 2023, the research team identified the HiDE‐I, a structured clinician‐administered tool developed by Teo et al. [24] and, following an internal review of its clinical relevance, formally integrated it into the protocol beginning January 1, 2024.

### 2.2. Participants

**FIGURE 1:**
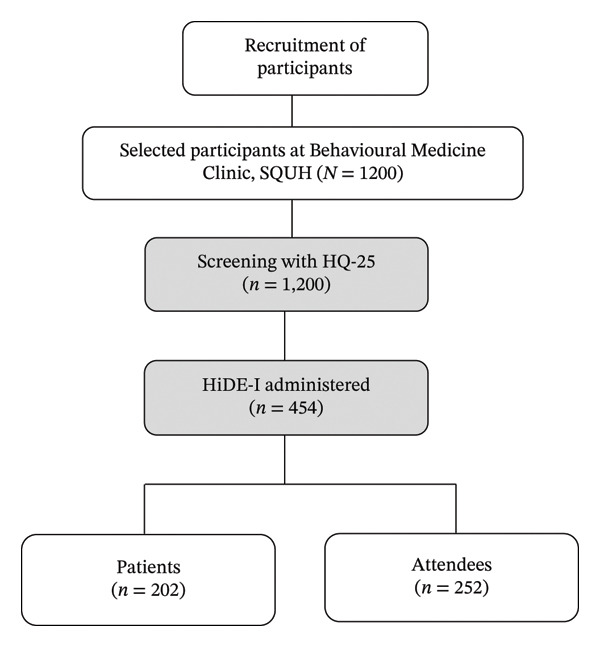
Flowchart of participant recruitment in the assessment of hikikomori at Behavioral Medicine Clinic, SQUH, Oman, 2025.

Participants included both patients attending the Behavioral Medicine Clinic and their companions (hereafter referred to as attendees) who were not themselves seeking clinical services. Eligible individuals were Omani adults aged 18–59 years who provided informed consent and completed the HQ‐25. Exclusion criteria included severe cognitive impairment or inability/refusal to provide informed consent. Beginning January 2024, all individuals present at the clinic—regardless of HQ‐25 score—were invited to participate in the HiDE‐I diagnostic assessment to classify hikikomori cases across three levels of diagnostic certainty: broad, strict, and confirmed. In total, 454 out of the 1200 participants completed both HQ‐25 and HiDE‐I assessments (see Figure 1). Of 1350 individuals approached, 1200 completed the HQ‐25, yielding an overall response rate of 88.9%. Reasons for nonparticipation included refusal to provide consent (7.1%) and incomplete assessments (4.0%).

### 2.3. Case Ascertainment

Hikikomori diagnoses were based on the HiDE‐I, which categorizes individuals into three clinical subtypes. Pathological hikikomori refers to individuals exhibiting social withdrawal lasting at least 6 months, accompanied by significant psychological distress or functional impairment. At‐risk hikikomori describes individuals with emerging social withdrawal symptoms who do not meet full diagnostic criteria. Hikikomori resemblance refers to individuals who display social withdrawal behaviors without clear evidence of distress or functional impairment. A total of 159 participants met the HiDE‐I criteria for one of these subtypes. Classifications were determined solely through the structured HiDE‐I interview, independent of HQ‐25 screening scores.

### 2.4. Case Definitions

Table 1 summarizes the hierarchical case definitions used in this study based on HQ‐25 screening thresholds and HiDE‐I diagnostic classifications.

**TABLE 1:** Overview of case definitions based on HiDE‐I criteria and HQ‐25 score cutoffs.

Features	Case definition	
	Strict HiDE‐I	Confirmed HiDE‐I
Purpose	Broader identification of clinically significant social withdrawal	Conservative identification of fully pathological cases
HiDE‐I criteria^a^	HiDE‐I classification as pathological, at‐risk, or resembling hikikomori	HiDE‐I classification as pathological
Diagnostic certainty	Moderate (dual confirmed)	High (clinically verified)

^a^In both case definitions based on HiDE‐I criteria, HQ‐25 criteria were applied based on variant cutoffs of HQ‐25 score as follows: score ≥ 42, ≥ 50, ≥ 62, or ≥ 75.

### 2.5. Case Definitions Based on HQ‐25

The HQ‐25 is a 25‐item self‐report measure assessing social withdrawal behaviors. In this study, four cutoff thresholds (≥ 42, ≥ 50, ≥ 62, and ≥ 75) were evaluated to classify participants into positive or negative cases at different sensitivity and specificity levels. Participants who scored at or above each respective threshold on the HQ‐25 were provisionally classified as positive cases, pending further validation through HiDE‐I structured interviews.

### 2.6. Case Definitions Based on HiDE‐I

To achieve greater diagnostic certainty, participant classifications were further refined using the HiDE‐I. Two tiers of diagnostic definitions were applied depending on the combination of HQ‐25 screening results and HiDE‐I clinical findings.

The strict HiDE‐I definition included participants who scored at or above the respective HQ‐25 threshold and were classified on the HiDE‐I as either pathological hikikomori, at‐risk hikikomori, or resembling hikikomori. This approach aimed to enhance specificity by requiring dual confirmation through both self‐report screening and clinical interview, capturing a wider spectrum of individuals demonstrating significant social withdrawal.

The confirmed HiDE‐I definition represented the most stringent case classification. It included participants who scored at or above the respective HQ‐25 cutoff and were diagnosed through the HiDE‐I specifically as pathological hikikomori, defined by persistent social withdrawal for at least six months accompanied by marked psychological distress or functional impairment. This definition provided the highest level of diagnostic certainty, isolating clinically verified cases.

The application of this two‐tiered framework across multiple HQ‐25 thresholds allowed for a more nuanced estimation of hikikomori prevalence, distinguishing between emerging withdrawal tendencies and fully pathological cases.

### 2.7. Study Tools

HQ‐25: The HQ‐25 is a 25‐item self‐report questionnaire designed to assess social withdrawal behaviors over the past six months (Teo et al.). A total score of 42 or higher indicates high risk for hikikomori. The validated Arabic version used in this study demonstrated excellent internal consistency (Cronbach’s alpha = 0.92; Al‐Sibani et al.). Multiple cutoff thresholds (≥ 42, ≥ 50, ≥ 62, ≥ 75) were tested against HiDE‐I classifications to optimize diagnostic accuracy. At each cutoff, participants scoring above the threshold were assumed to be “at risk” of hikikomori based on the screening tool, pending validation through structured clinical assessment.

HiDE‐I: The HiDE‐I is a structured clinical interview developed by Kato et al. to categorize individuals into three diagnostic categories: resembling, at‐risk, or pathological hikikomori. It evaluates core features such as physical withdrawal, functional impairment, and duration of social isolation. Research assistants received standardized training to administer the HiDE‐I, supported by materials from the official Hikikomori Lab website. Sociodemographic Questionnaire: Participants provided information on age, gender, marital status, educational level, income, employment status, and governorate of residence.

## 3. Ethical Considerations

Ethical approval was obtained from the Sultan Qaboos University Hospital Ethics Committee (MREC #2260). Written informed consent was secured from all participants prior to enrollment. Data confidentiality was strictly maintained through anonymization and secure storage. All study procedures adhered to the principles outlined in the Declaration of Helsinki. Clinical trial registration: Not applicable.

## 4. Statistical Analysis

All analyses were conducted using SPSS version 27.0 (IBM Corp., Armonk, NY, USA). Chi‐square tests were used to examine associations between HQ‐25 screening results and HiDE‐I diagnoses. Diagnostic performance was evaluated at multiple HQ‐25 cutoff thresholds (≥ 42, ≥ 50, ≥ 62, and ≥ 75) against the two diagnostic definitions: the strict HiDE‐I and confirmed HiDE‐I category. For each threshold and case definition, we calculated sensitivity, specificity, positive predictive value (PPV), negative predictive value (NPV), and likelihood ratios (LR+ and LR−). ROC curve analyses were used to assess classification accuracy, with area under the curve (AUC) values reported. This approach follows standard practices in psychiatric diagnostic validation [3, 26, 28, 29].

## 5. Results

### 5.1. Sociodemographic Characteristics

Table 2 presents the sociodemographic characteristics of the total sample (*N* = 454), including patients (*n* = 202) and attendees (*n* = 252). No significant demographic or clinical differences were observed between participants recruited before and after the introduction of HiDE‐I in January 2024. The majority of participants were female (63.9%), aged 18–29 years (52.0%), and unemployed (68.3%). A significantly higher proportion of patients were female (69.3%) compared to attendees (59.9%) (*p* = 0.048). Age distribution also differed significantly across groups, with 62.9% of patients aged 18–29 compared to 43.3% of attendees (*p* = 0.001). Employment status (*p* = 0.001), marital status (*p* = 0.001), and parental status (*p* = 0.001) also showed statistically significant group differences. No significant differences were observed by family income (*p* = 0.115), education years (*p* = 0.895), or governorate (*p* = 0.328).

**TABLE 2:** Sociodemographic characteristics of participants (*N* = 454) recruited from the Behavioral Medicine Clinic, SQUH, Oman, 2025.

Characteristic	All participants (*N* = 454)		Patients (*N* = 202)		Attendees (*N* = 252)		*p* value
	N	%	N	%	N	%	
Gender							0.04
Female	290	63.9	140	69.3	151	59.9	
Male	164	36.1	62	30.7	101	40.1	
Age categories							0.001
18–29	236	52.0	127	62.9	109	43.3	
30–39	108	23.8	39	19.3	69	27.4	
40–49	88	19.4	30	14.9	58	23.0	
50–59	20	4.4	5	2.5	15	6.0	
> 60	2	0.4	1	0.5	1	0.4	
Family income							0.11
Less than 1000	214	47.1	122	60.4	122	48.4	
1000–1999	153	33.7	67	33.2	86	34.1	
2000–2999	51	11.2	28	13.9	27	10.7	
3000 or more	36	7.9	30	14.9	17	6.7	
Employment							0.001
Employed	144	31.7	43	21.3	90	35.7	
Unemployed	310	68.3	159	78.7	162	64.3	
Marital Status							0.001
Never married	246	54.2	128	63.4	118	46.8	
Ever married	208	45.8	74	36.6	134	53.2	
Have children							0.001
Yes	175	38.5	60	29.7	115	45.6	
No	279	61.5	142	70.3	137	54.4	
Education years							0.89
Less than 12 years	17	3.7	7	3,5	10	4.0	
12 years	125	27.5	54	26.7	71	28.2	
Above 12 years	312	68.7	141	69.8	171	67.9	
Governorate							0.32
Muscat	207	45.6	105	52.0	102	40.5	
Batinah	133	29.3	56	27.7	77	30.6	
Dakhiliyah	65	14.3	22	10.9	43	17.1	
Sharqiyah	29	6.4	18	8.9	18	7.1	
Dhahirah	15	3.3	8	4.0	8	3.2	
Dhofar	3	0.7	2	1.0	2	0.8	
Buraimi	2	0.4	2	1.0	2	0.8	

### 5.2. Diagnostic Performance of HQ‐25 Across Cutoff Thresholds

Table 3 shows the diagnostic performance of HQ‐25 at the ≥ 42 cutoff. Among all participants, sensitivity and specificity for detecting strict HiDE‐I cases were 62.9% and 57.6%, respectively (AUC = 0.593), while confirmed HiDE‐I classifications yielded higher sensitivity (80.0%) and lower specificity (53.4%) with an AUC of 0.554. Patients showed higher sensitivity (69.4%) but lower specificity (46.2%) under strict HiDE‐I criteria (AUC = 0.575). Attendees had the lowest AUC for confirmed HiDE‐I cases (0.522).

**TABLE 3:** Sensitivity, specificity, and AUC values for HQ‐25 ≥ 42 across study groups, stratified by strict and confirmed definitions of HiDE, Oman, 2025.

HQ‐25 cutoffs	All participants (*N* = 454)		Patients (*N* = 202)		Attendees (*N* = 252)	
	Strict	Confirmed	Strict	Confirmed	Strict	Confirmed
Most sensitive (HQ‐25 ≥ 42)						
Total HQ‐25 cases	225	225	122	122	100	100
Total HQ‐25 noncases	229	229	80	80	152	152
Total HiDE‐I cases	159	40	85	31	74	9
Total HiDE‐I noncases	295	414	117	171	178	243
True positive	100	32	59	27	41	5
True negative	170	221	54	76	119	83
False positive	125	193	63	95	59	95
False negative	59	8	26	4	33	69
Sensitivity (95% CI)	62.9 (54.9–70.4)	80.0 (64.4–90.9))	69.4 (58.1–79.2)	87.1 (70.2–96.4)	55.4 (43.6–66.8)	6.8 (2.2–15.1)
Specificity (95% CI)	57.6 (51.8–63.2)	53.4 (48.4–58.3)	46.2 (36.5–56.2)	44.4 (36.7–52.3)	66.9 (59.1–74.1)	46.6 (39.1–54.2)
Predictive value (+ve)	44.4	14.2	48.4	22.1	41.4	5.0
Predictive value (‐ve)	74.2	96.5	67.5	95.0	78.3	54.6
Accuracy	59.5	55.7	56.4	51.0	63.5	34.9
Likelihood ratio (+ve)	1.48	1.716	1.289	1.567	1.674	0.106
Likelihood ratio (‐ve)	0.64	0.374	0.662	0.291	0.668	0.781
Area under curve (AUC) (95% CI)	0.593 (0.54–0.65)	0.554 (0.48–0.63)	0.575 (0.49–0.66)	0.651 (0.55–0.74)	0.584 (0.50–0.67)	0.522 (0.43–0.61)

Table 4 presents the results for the HQ‐25 ≥ 50 cutoff. Sensitivity decreased and specificity improved compared to the ≥ 42 threshold. Among all participants, the AUC was 0.620 for strict HiDE‐I and 0.790 for confirmed HiDE‐I cases. Patients showed improved diagnostic precision (AUC = 0.660 for strict; 0.810 for confirmed), while attendees had moderate performance (AUC = 0.580 and 0.750 for strict and confirmed, respectively).

**TABLE 4:** Sensitivity, specificity, and AUC values for HQ‐25 ≥ 50 across study groups, stratified by strict and confirmed definitions of HiDE, Oman, 2025.

HQ‐25 cutoffs	All participants (*N* = 454)		Patients (*N* = 202)		Attendees (*N* = 252)	
	Strict	Confirmed	Strict	Confirmed	Strict	Confirmed
Most sensitive (HQ‐25 ≥ 50)						
Total HQ‐25 cases	137	137	85	85	52	52
Total HQ‐25 noncases	317	317	117	117	200	200
Total HiDE‐I cases	159	40	85	31	74	9
Total HiDE‐I noncases	295	414	117	171	178	243
True positive	70	26	47	22	23	4
True negative	228	303	79	108	149	195
False positive	67	111	38	63	29	48
False negative	89	14	38	9	51	5
Sensitivity (95% CI)	44.0 (36.2–52.1)	65.0 (48.3–79.4)	55.3 (44.1–66.1)	71.0 (51.9–85.8)	31.1 (20.8–42.9)	44.4 (13.7–78.0)
Specificity (95% CI)	77.5 (72.1–81.9)	73.2 (68.6–77.4)	67.5 (58.2–75.9)	63.2 (55.5–70.4)	83.7 (77.4–88.8)	80.2 (74.7–85.1)
Predictive value (+ve)	51.1	19.0	55.3	25.9	44.2	7.7
Predictive value (‐ve)	71.9	95.6	67.5	92.3	74.5	97.5
Accuracy	65.6	72.7	62.4	64.4	68.3	78.6
Likelihood ratio (+ve)	1.956	2.426	1.702	1.93	1.912	2.242
Likelihood ratio (‐ve)	0.723	0.478	0.661	0.458	0.822	0.693
Area under curve (AUC) (95% CI)	0.62 (0.58–0.67)	0.79 (0.75–0.83)	0.66 (0.62–0.70)	0.81 (0.77–0.85)	0.58 (0.53–0.63)	0.75 (0.71–0.79)

Table 5 displays results for the ≥ 62 cutoff, showing a marked decline in sensitivity and an increase in specificity. For all participants, sensitivity and specificity for strict HiDE‐I were 13.8% and 94.9%, respectively (AUC = 0.620). Confirmed HiDE‐I yielded an AUC of 0.790. Patients demonstrated slightly higher AUCs (0.660 for strict, 0.820 for confirmed), while attendees again had lower sensitivity but high specificity (AUC = 0.590 and 0.720, respectively).

**TABLE 5:** Sensitivity, specificity, and AUC values for HQ‐25 ≥ 62 across study groups, stratified by strict and confirmed definitions of HiDE, Oman, 2025.

HQ‐25 cutoffs	All participants (*N* = 454)		Patients (*N* = 202)		Attendees (*N* = 252)	
	Strict	Confirmed	Strict	Confirmed	Strict	Confirmed
Most sensitive (HQ‐25 ≥ 62)						
Total HQ‐25 cases	37	37	27	27	10	10
Total HQ‐25 noncases	417	417	175	175	242	242
Total HiDE‐I cases	159	40	85	31	74	9
Total HiDE‐I noncases	295	414	117	171	178	243
True positive	22	12	17	11	5	1
True negative	280	389	107	155	173	234
False positive	15	25	10	16	5	9
False negative	137	28	68	20	69	8
Sensitivity (95% CI)	13.8 (9.3–20.1)	30.0 (18.1–45.4)	20.0 (12.9–29.7)	35.5 (21.1–53.1)	6.8 (2.9–14.9)	11.1 (2.0–43.5)
Specificity (95% CI)	94.9 (91.8–96.9)	93.9 (91.2–95.9)	91.5 85.0–95.3)	90.6 (85.3–94.2)	97.2 (93.6–98.8)	96.3 (93.1–98.0)
Predictive value (+ve)	59.5	32.4	63.0	40.7	50.0	10.0
Predictive value (‐ve)	67.2	93.3	61.1	88.6	71.5	96.7
Accuracy	66.5	88.2	61.4	82.2	70.6	93.3
Likelihood ratio (+ve)	2.716	4.938	2.356	3.774	2.429	3.0
Likelihood ratio (‐ve)	0.905	0.745	0.874	0.712	0.958	0.923
Area under curve (AUC) (95% CI)	0.62 (0.56–0.68)	0.79 (0.74–0.84)	0.66 (0.59–0.73)	0.82 (0.76–0.87)	0.59 (0.53–0.65)	0.72 (0.66–0.78)

Table 6 summarizes the most conservative cutoff (≥ 75). Sensitivity was the lowest across all groups, ranging from 0.0% to 4.7% (strict) and up to 12.9% (confirmed, patients), while specificity exceeded 99%. The AUC was 0.580 for all participants under strict criteria, 0.620 for patients, and 0.550 for attendees. Under confirmed HiDE‐I criteria, AUC values were 0.790 for all participants, 0.850 for patients, and 0.760 for attendees.

**TABLE 6:** Sensitivity, specificity, and AUC values for HQ‐25 ≥ 75 across study groups, stratified by strict and confirmed definitions of HiDE, Oman, 2025.

HQ‐25 cutoffs	All participants (*N* = 454)		Patients (*N* = 202)		Attendees (*N* = 252)	
	Strict	Confirmed	Strict	Confirmed	Strict	Confirmed
Most sensitive (HQ‐25 ≥ 75)						
Total HQ‐25 cases	6	6	5	5	1	1
Total HQ‐25 noncases	448	448	197	197	251	251
Total HiDE‐I cases	159	40	85	31	74	9
Total HiDE‐I noncases	295	414	117	171	178	243
True positive	4	4	4	4	0	0
True negative	293	412	116	197	177	242
False positive	2	2	1	1	1	1
False negative	155	36	81	27	74	9
Sensitivity (95% CI)	2.5 (1.0–6.3)	10.0 (4.0–23.1)	4.7 (1.8–11.5)	12.9 (5.1–28.9)	0.0 (‐0.0–4.9)	0.0 (‐0.0–4.9)
Specificity (95% CI)	99.3 (97.6–99.8)	99.5 (98.3–99.9)	99.1 (95.3–99.8)	99.5 (97.2–99.9)	99.4 (96.9–99.9)	99.6 (97.7–99.9)
Predictive value (+ve)	66.7	66.7	80.0	80.0	0.0	0.0
Predictive value (‐ve)	65.4	92.0	58.9	88.0	70.5	96.4
Accuracy	91.6	91.6	99.0	99.0	98.4	98.4
Likelihood ratio (+ve)	3.571	20.0	5.222	25.8	0.0	0.0
Likelihood ratio (‐ve)	0.979	0.904	0.961	0.875	1.006	1.004
Area under curve (AUC) (95% CI)	0.58 (0.52–0.64)	0.79 (0.70–0.88)	0.62 (0.54–0.70)	0.85 (0.76–0.94)	0.55 (0.47–0.63)	0.76 (0.57–0.95)

## 6. Discussion

To our knowledge, this is the first study in Oman and the broader Arab region to evaluate the diagnostic validity of the HQ‐25 using the clinician‐administered HiDE‐I as a reference standard. While previous research validated the HQ‐25 in nonclinical Omani populations [8, 18], our study extends its applicability to psychiatric settings. Our ROC curve analyses were used to assess classification accuracy and explore optimal cutoff thresholds within a culturally distinct context.

In our study, the standard HQ‐25 cutoff, as originally proposed by Teo et al. [14], offered reasonable sensitivity but limited specificity. This pattern is consistent with studies conducted in Spain and China [12, 30], where lower thresholds were associated with overidentification, particularly in nonclinical groups. In our sample, higher thresholds improved specificity and PPV but reduced sensitivity.

Our findings echo those of Hajek [31], who reported that prevalence dropped when more conservative cutoffs were applied. These results support previous calls to tailor cutoff thresholds to specific populations and clinical settings [4, 32]. Stratified ROC analyses from our study showed better HQ‐25 performance among psychiatric patients than community attendees, particularly at higher thresholds. These findings parallel limitations noted in other validation studies, which highlight the risk of overidentification when the HQ‐25 is used in isolation [31].

### 6.1. Diagnostic Alignment and the Role of HiDE‐I

Our data showed that the HiDE‐I provided a more nuanced classification system by identifying pathological, at‐risk, and resembling subtypes. This multidimensional approach aligns with recent conceptualizations of hikikomori as a spectrum rather than a binary condition [11, 24]. A substantial proportion of our participants fell into these transitional categories, supporting stepped‐care intervention models [33].

Not all individuals who screened positive on the HQ‐25 in our sample were confirmed as hikikomori via HiDE‐I, and some clinically confirmed cases were missed by the HQ‐25. These misclassification patterns align with earlier research [18, 31], reinforcing the HQ‐25’s limitations as a standalone diagnostic tool.

### 6.2. Misclassification and Screening Limitations

Our findings indicate that the HQ‐25’s screening performance varied substantially across both cutoff thresholds and participant subgroups. In the community sample, lower thresholds were more likely to yield false positives, while higher thresholds tended to miss individuals with clinically significant withdrawal. Among psychiatric patients, the HQ‐25 showed relatively stronger diagnostic alignment with HiDE‐I classifications. These observations reinforce concerns raised in recent validation studies about the limitations of using the HQ‐25 as a standalone tool [31].

### 6.3. HiDE‐I and Multilevel Case Differentiation

This study is the first in the Middle East to simultaneously apply the HQ‐25 and HiDE‐I, enabling a more layered approach to case identification. Our findings reinforce the utility of the HQ‐25 for initial screening while highlighting its tendency to overidentify cases at lower thresholds and underidentify them at higher ones in the absence of structured clinical interviews. The HiDE‐I’s ability to differentiate among pathological, at‐risk, and resembling hikikomori [11, 24] was critical in our sample, where a substantial proportion of cases fell into transitional or subclinical categories that would have been missed or misclassified using HQ‐25 alone. Notably, many participants who screened positive on the HQ‐25 did not meet diagnostic criteria on the HiDE‐I, while others who were confirmed cases by HiDE‐I were not captured by the questionnaire. These discrepancies emphasize the importance of integrating multilevel assessment strategies to improve diagnostic accuracy.

In our study, the HiDE‐I enabled identification of nuanced subtypes within both clinical and community settings, which proved especially valuable given the variation in performance of the HQ‐25 across groups. Aligning diagnostic certainty with sample characteristics enhanced the reliability of our prevalence estimates and clarified the spectrum of social withdrawal severity [4]. Given hikikomori’s increasing recognition as a global mental health concern, culturally sensitive frameworks such as the HiDE‐I—which emphasize functional impairment, psychosocial distress, and withdrawal severity—are essential for accurate diagnosis in diverse sociocultural contexts [4, 14].

### 6.4. ROC Analysis and Optimal Cutoff Selection

ROC analyses provided additional insights into how cutoff thresholds influence diagnostic classification. Higher thresholds yielded greater specificity but reduced case detection, particularly in nonclinical subgroups. In contrast, moderate thresholds offered a more balanced profile for screening. These results support prior recommendations that ROC findings be supplemented with clinical interviews to improve diagnostic accuracy and reduce the risks of over‐ or underclassification [3, 24, 26].

Stratified analyses also confirmed that the HQ‐25 performed most consistently among psychiatric patients, where symptom severity is expected to be more pronounced. In community samples, AUC values were lower and PPV was limited, especially at more liberal thresholds. These results further highlight the risks of relying exclusively on self‐report measures in nonclinical settings [18, 31].

### 6.5. Sociodemographic Patterns in Hikikomori Diagnosis

Our study identified several significant demographic differences between patients and community attendees, with patients more likely to be younger, female, unemployed, unmarried, and childless. Hikikomori cases in our sample were more common among women and those from higher‐income households, contrasting with findings from Japan, where cases are often reported among low‐income men [4]. Gender‐based help‐seeking behaviors may explain this discrepancy [34, 35], and studies in Slovakia and Singapore report that socially withdrawn women often experience greater distress yet access services more readily [36, 37]. Cultural norms in Japan may normalize female withdrawal in domestic roles, leading to underrecognition [38, 39]. In Oman, however, stricter social expectations for women and greater openness to seeking psychological support may have contributed to their overrepresentation in our clinic‐based sample.

Our findings align with the literature showing hikikomori across genders and age groups, challenging the view that it is male‐dominated or youth‐specific [18, 40, 41]. Cultural and socioeconomic context appears to shape its presentation [6, 8, 12, 30]. Consistent with earlier studies, cases were more frequently identified in psychiatric settings, highlighting the gap between treatment‐seeking individuals and undiagnosed populations [35, 42, 43]. While older men are more affected in Japan due to social isolation and economic pressures [32, 44], our data suggest cultural expectations and financial dependence may increase identification of young women in Oman.

Although not statistically significant, most identified cases were in the 18–29 age group, supporting global findings that link early adulthood to social withdrawal risk [4, 42]. Digital dependency and postpandemic uncertainty may contribute to this trend [2, 45]. The education level was also associated with the hikikomori risk, consistent with studies linking academic stress, identity issues, and career anxiety to withdrawal [22, 23, 41, 46].

These findings highlight the need for regionally adapted screening and intervention strategies that reflect gender, cultural norms, and socioeconomic context [35, 36, 40, 44].

### 6.6. Subgroup Differentiation and Clinical Implications

One of the novel contributions of this study is the use of HiDE‐I to categorize hikikomori into pathological, at‐risk, and resembling subgroups. These distinctions are essential for early intervention, as supported by recent findings showing that individuals in early‐stage hikikomori (< 3 months) already exhibit significant distress and elevated gaming disorder symptoms [33]. Kato et al. [11] similarly argue for a paradigm shift in which physical isolation is not pathologized unless accompanied by distress or impairment. Our data support this: a significant proportion of individuals classified as “resembling” or “at‐risk” still showed functional challenges, justifying the need for a stepped‐care model of intervention.

### 6.7. Implications for Diagnosis in a Postpandemic Context

The importance of distinguishing between pathological and nonpathological hikikomori has been emphasized in the recent literature, especially considering lifestyle changes post‐COVID‐19 [11]. People working remotely or staying home are not necessarily socially impaired. HiDE‐I’s inclusion of distress and functional impairment criteria offers a more grounded diagnostic framework in today’s “new normal.” As Kubo et al. [33] argue, early pathological cases (within 3 months) can already show high psychiatric comorbidity and gaming disorder, reinforcing the need for early, nuanced intervention.

### 6.8. Limitations

This study has several limitations. First, while the HQ‐25 is a validated screening tool, it is not diagnostic. To address this, we incorporated the HiDE‐I structured clinical interview from January 2024 onward. Comparisons indicated no systematic demographic or clinical differences between pre‐ and postintegration participants, reducing the likelihood of bias from this procedural change. As the current reference standard for hikikomori diagnosis, the HiDE‐I enabled a multitiered classification into pathological, at‐risk, and resembling subtypes, improving diagnostic specificity and reducing false positives. However, it is important to note that in our earlier prevalence study on hikikomori in Oman (unpublished), we conducted inter‐rater agreement testing between assessors and found satisfactory reliability in the application of the HiDE‐I. While the present study did not repeat this exercise, those findings provide some reassurance about assessor consistency and reliability. Second, the cross‐sectional design limits causal inference. However, this study was originally nested in a cross‐sectional study with a nested case–control component to enhance subgroup analysis and internal comparisons between clinical and nonclinical groups. Third, the sample was drawn from a single behavioral medicine clinic, which may limit generalizability. To mitigate this, we included both psychiatric patients and community attendees to increase heterogeneity and allow for stratified analysis. Fourth, while our findings suggested a higher proportion of women meeting hikikomori criteria, this may partly reflect sampling and help‐seeking patterns rather than true prevalence. Additional qualitative or mixed‐method research is needed to clarify gendered pathways into care in Oman. Finally, our selection of thresholds was informed by diagnostic performance indices (sensitivity, specificity, and AUC). At the same time, we recognize that cultural validation is equally important. The way social withdrawal is understood and reported can vary across cultures, particularly in terms of stigma, help‐seeking behavior, and symptom endorsement. For this reason, the thresholds identified in this Omani sample should be interpreted with caution until confirmed in future cross‐cultural validation studies.

### 6.9. Interventions and Future Research

Addressing loneliness remains key in mitigating hikikomori risk, as it strongly correlates with social withdrawal [13]. Hajek [31] confirmed this association in a representative German sample, emphasizing its relevance across contexts.

Future research should explore psychiatric comorbidities [11, 37] and refine screening tools by incorporating behavioral and biometric data [11, 14]. Longitudinal studies are also needed to trace transitions between hikikomori subtypes over time, especially in non‐Western populations.

### 6.10. Conclusion

This study supports the HQ‐25 as a useful screening tool but emphasizes the importance of structured assessments such as the HiDE‐I for diagnostic confirmation. The HiDE‐I’s subclassification framework enhances clinical decision‐making by differentiating transient, at‐risk, and pathological withdrawal. ROC findings highlight the need for culturally calibrated cutoff scores. Collectively, these findings underscore the importance of regionally adapted diagnostic tools and early intervention models tailored to psychosocial and cultural contexts.

**Nomenclature**
AUC: Area under the curve
HiDE‐I: Hikikomori Diagnostic Evaluation Interview
HLID: Hikikomori‐Like Idiom of Distress
HQ‐25: Hikikomori Questionnaire‐25
LR+: Positive likelihood ratio
LR−: Negative likelihood ratio
NPV: Negative predictive value
PPV: Positive predictive value
ROC: Receiver operating characteristic
SQUH: Sultan Qaboos University Hospital

## Author Contributions

Nutaila Al‐Kharusi conceptualized the study, led data collection and analysis, and drafted the manuscript. Yahya M. Al‐Farsi supervised the research process and reviewed all analyses. Moon Fai Chan assisted with statistical analysis. Samir Al‐Adawi and Nasser Al‐Sibani provided clinical oversight and recruitment.

Nutaila Al‐Kharusi is a PhD candidate in Public Health and Epidemiology in Sultan Qaboos University, Oman.

## Funding

This study was supported by the Department of Family Medicine and Public Health and Department of Behavioral Medicine in the College of Medicine and Health Sciences, Sultan Qaboos University.

## Disclosure

All authors read and approved the final manuscript.

## Ethics Statement

Ethical approval was obtained from the Sultan Qaboos University Hospital Ethics Committee (MREC #2260). Written informed consent was obtained from all participants.

## Consent

Please see Ethics Statement.

## Conflicts of Interest

The authors declare no conflicts of interest.

## Data Availability

The datasets generated and/or analyzed during the current study are available from the corresponding author on reasonable request.
